# A Case Report on Dyke-Davidoff-Masson Syndrome: A Rare Cause of Hemiparesis

**DOI:** 10.7759/cureus.34637

**Published:** 2023-02-04

**Authors:** Suvarna S Ganvir, Simran A Mishra, Maheshwari Harishchandre, Akhilendra B Khare, Shyam D Ganvir

**Affiliations:** 1 Neurophysiotherapy, Dr. Vithalrao Vikhe Patil Foundation's College of Physiotherapy, Ahmednagar, IND; 2 Internal Medicine, Dr. Vithalrao Vikhe Patil Foundation's Medical College and Hospital, Ahmednagar, IND; 3 Community Physiotherapy, Dr. Vithalrao Vikhe Patil Foundation's College of Physiotherapy, Ahmednagar, IND

**Keywords:** physiotherapy, cerebral hemiatrophy, ddms, hemiparesis, dyke davidoff masson syndrome

## Abstract

Infantile hemiparesis resulting from Dyke-Davidoff-Masson syndrome (DDMS) is an uncommon condition, especially in patients with no positive natal history. The age of presentation is dependent on when the neurologic insult occurred, and distinctive alterations may not appear until puberty. The left hemisphere and the male gender are more frequently involved. Common findings that can be seen are seizures, hemiparesis, mental retardation, and facial changes. Characteristic MRI findings are dilation of the lateral ventricles, hemiatrophy of the cerebrum, frontal sinus hyperpneumatization, and compensatory hypertrophy of the skull. Here, we report a 17-year-old female patient who reported physiotherapy treatment after the attack of epilepsy, with the complaint of inability to use the right hand for functional activities and gait deviations. Patient examination revealed typical chronic hemiparesis of the right side with mild cognitive affection. Brain investigation confirms the diagnosis of DDMS.

## Introduction

Dyke-Davidoff-Masson syndrome (DDMS) was first described by Dyke, Davidoff, and Mason in 1933 in the form of a case series of nine patients [1]. The clinical characteristics of these patients included hemiparesis, seizures, facial asymmetry, and mental retardation [2]. Radiologically, there is cerebral hemiatrophy, dilation of lateral ventricles, frontal sinus hyperpneumatization, and compensatory hypertrophy of the skull [3] caused by cerebral insult in utero or during early life due to brain damage.

Depending on when the neurologic insult occurs, the age at which symptoms arise significantly changes, and symptoms may not show up until puberty. The male gender and the left hemisphere are more frequently involved. Since 1933, 100 cases have been reported in the literature, with only 21 cases in adults [4]. However, the age at which patients report to healthcare facilities has varied in the literature, as have the symptoms present. Here, we describe a 17-year-old female who was referred for physiotherapy treatment with a chief complaint of infantile hemiparesis, two episodes of seizure on one day at the age of 17, and a medical diagnosis of DDMS.

## Case presentation

A 17-year-old female was brought to the hospital with a complaint of two episodes of sudden onset seizures at an interval of approximately six to seven hours, one in the morning and one in the afternoon. The mother witnessed it and mentioned that the patient was producing choking sounds during sleep (both times it occurred during sleep). The mother mentioned that the patient was completely still, eyes open, staring in one direction, with no jerky movements, no rolling of the eyeball, no drooling of saliva, and no biting of the tongue. It lasted approximately five to six minutes. The patient is not able to recall both episodes.

Birth history was not significant. However, the patient had a similar episode 14 years ago; the mother could not recall the exact history but mentioned that the patient developed weakness on the right side of the body and was unable to perform any movement with the right upper limb (UL) and lower limb (LL) a few hours after the episode. She had achieved all developmental milestones for her specific age before the episode, normally. No investigations were done at that time, and she was given Ayurvedic treatment. The patient attended school until the 7th grade and had learning difficulties, according to the mother. She had a past medical history of severe anaemia secondary to chronic malaria four years ago. The patient was admitted to the hospital, and a brain MRI was done, which led to the diagnosis of DDMS. She has four siblings - one elder sister, two younger sisters, and one younger brother - with no similar complaints.

**Figure 1:**
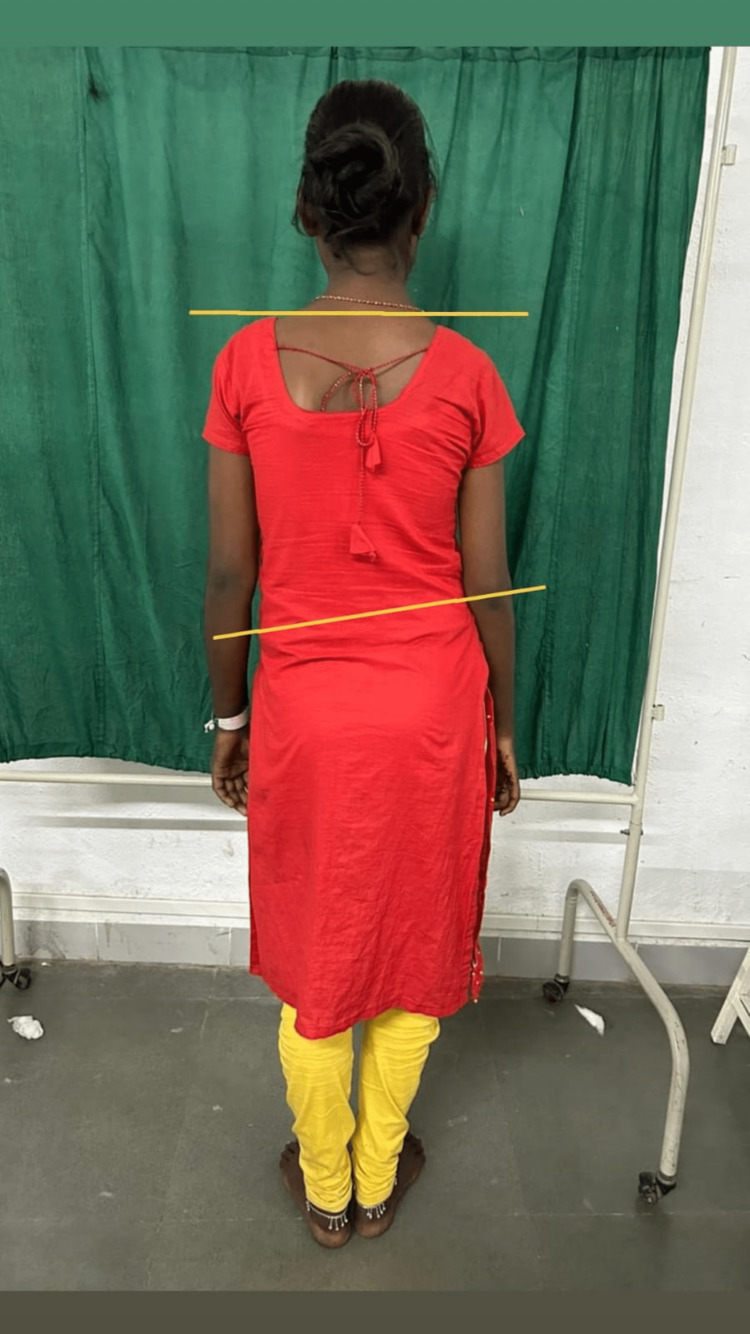
Mild scoliosis with hip hike on right side.

**Figure 2:**
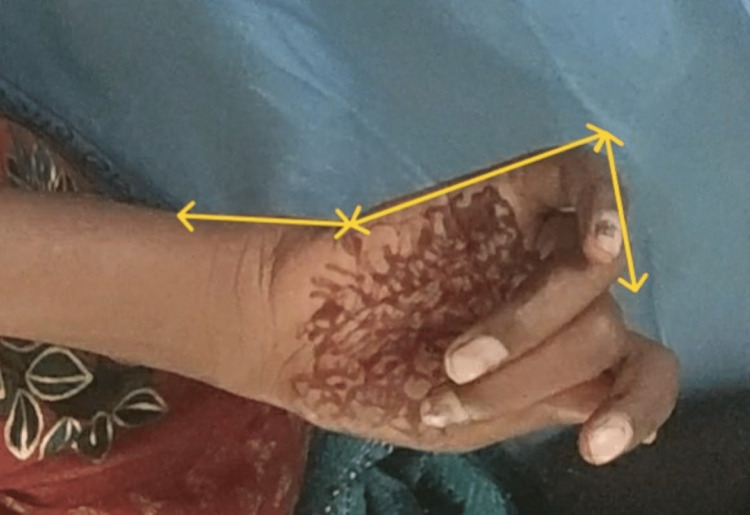
Attempt to make fist push wrist in extension with disproportionate finger flexion.

**Figure 3:**
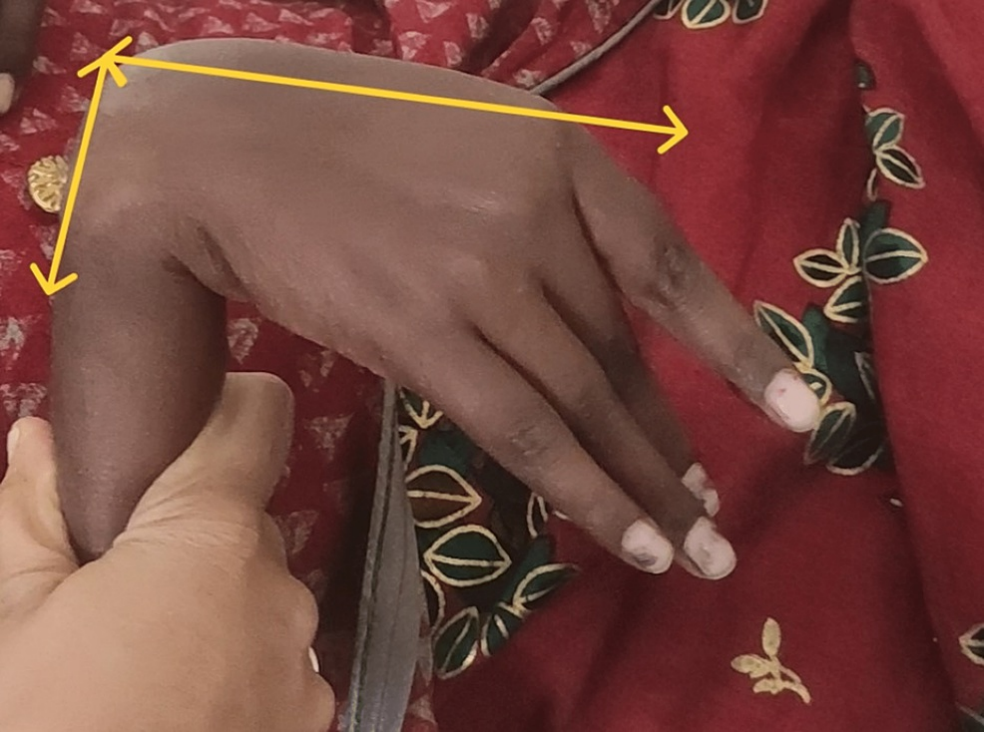
Tenodesis position of wrist and hand.

On observation, the patient appeared undernourished, and generalized muscle wasting could be appreciated in the right upper limb. She was underweight; her BMI was 16.9. The patient had difficulty paying attention during the examination, and the calculated MMSE score was 24, which is in the 25th percentile for her age and level of education [5]. The sensory examination was normal. Motor examination revealed Grade 1 spasticity on MAS in the right knee flexors and right wrist flexors. Muscle strength according to MRC grading is mentioned in Table 1. The strength of the abdominal muscles was poor, and the back extensor muscles were fair. All deep tendon reflexes were normal except the right patellar; the brisk and plantar response on the right side was extensor. Coordination testing revealed that movements were smooth but inaccurate and not controlled for the right upper and lower limbs. She had visible muscle atrophy in the right upper limb; girth measurement revealed a difference of 4 cm between the right and left arm and forearm. She had mild tightness of the right hamstring and tendoachilles and moderate tightness of the right hip adductors and investors. Postural examination revealed depression of the right shoulder compared to the left (Figure 1); the presence of mild scoliosis with concavity to the right side (Figure 1) increased lumbar lordosis in the right pelvic hike (Figure 1); and the hip externally rotated. For hand functions, her grasps and grip were poor, and a tenodesis grasp was present (Figures 2-3). Functionally, the patient was able to sit and walk independently with moderate deviation; the pelvis dropped to the left during the stance phase; hip flexion was reduced and went into external rotation at the time of acceleration; the right knee went into hyperextension; and heel strike was absent with an increased degree of toe out. She was able to perform all activities of daily livings (ADLs) with compensation of the left side in the upper extremity.

**Table 1:** Description of different muscle group strength/VCG in UL and LL. UL: upper limb, LL: lower limb, VCG: voluntary control grading.

Muscle group UL	Right	Left	Muscle group LL	Right	Left
Shoulder			Hip		
Flexors	3+/5	4+/5	Flexors	3+/5	4+/5
Extensors	3/5	4+/5	Extensors	3+/5	4/5
Abductors	3+/5	4+/5	Abductors	3+/5	4/5
Adductors	3+/5	4+/5	Adductors	3/5	4+/5
Elbow			Knee		
Flexors	4/5	5/5	Flexors	VCG-4	4+/5
Extensors	3+/5	4+/5	Extensors	VCG-5	4+/5
Wrist			Ankle		
Flexors	VCG-1	4+/5	Plantarflexors	2/5	4/5
Extensors	VCG-0	4+/5	Dorsiflexors	1/5	4/5
Finger			Toes		
Flexors	1/5	4+/5	Flexors	3/5	4/5
Extensors	0/5	4+/5	Extensors	3/5	4/5

**Figure 4:**
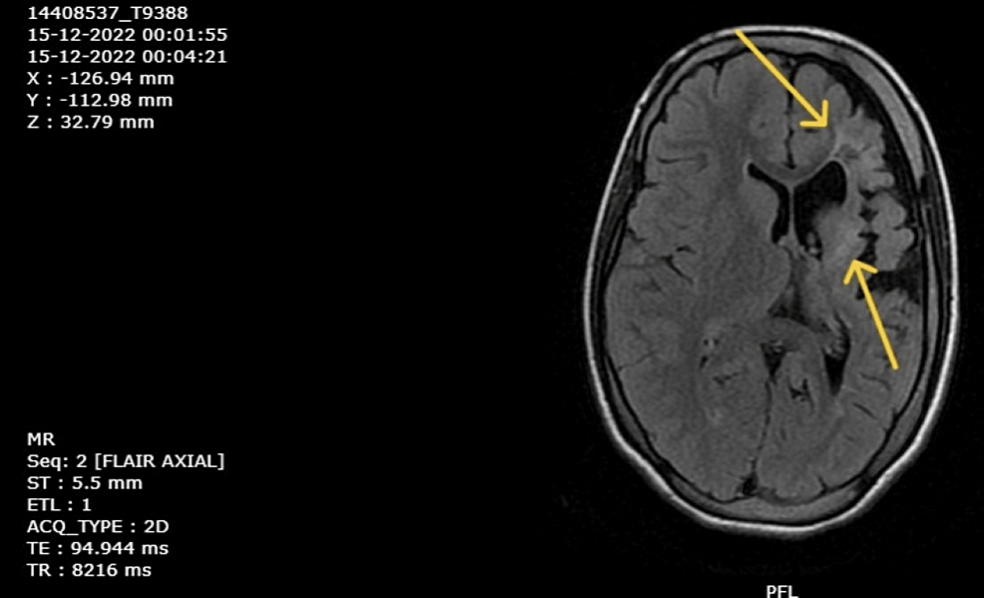
MRI scan of brain showing gliotic areas (December 10, 2022).

**Figure 5:**
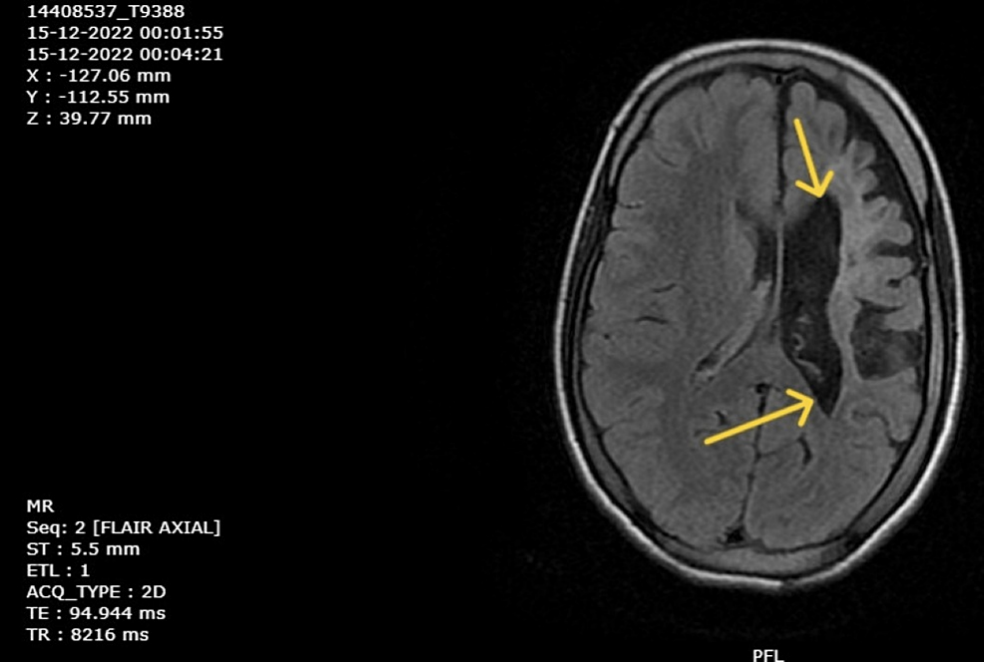
MRI of brain showing dilation of left lateral ventricle (December 10, 2022).

**Figure 6:**
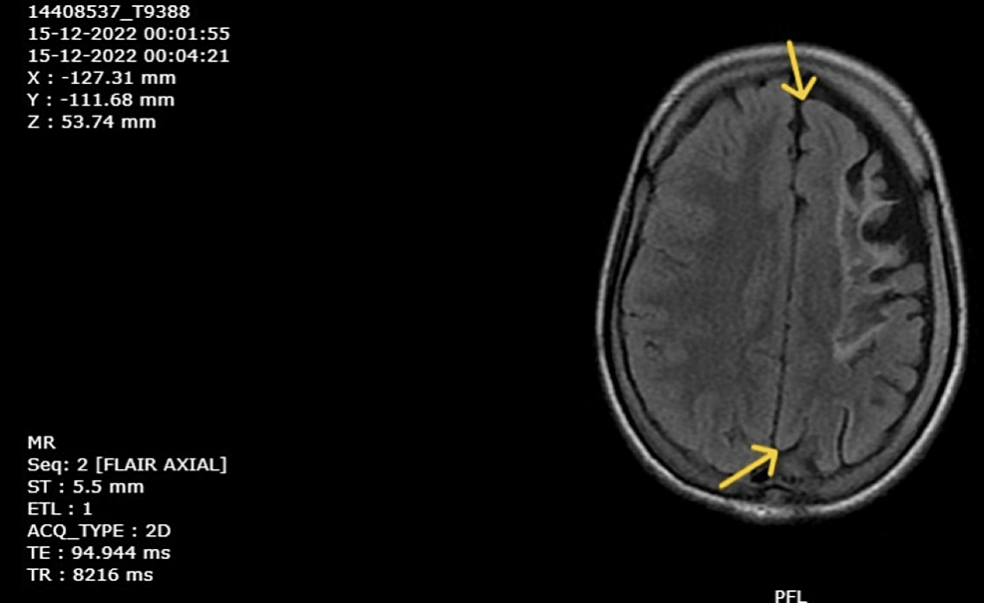
MRI of brain showing, paradoxical midline shift towards left side (December 10, 2022).

Magnetic resonance imaging (MRI) was done first on September 15, 2018, and reports were retrieved that suggested left cerebral hemiatrophy with gliotic changes in the left frontal, temporal, and parietal lobes along with atrophy of the midbrain and pons on the left side. Haematological investigations were done on December 9, 2022, and a complete blood count revealed moderate anemia. Renal and liver function tests were normal. An MRI of the brain was repeated on December 10, 2022, which revealed gliotic areas involving the left frontoparietal and temporal regions (Figure 4), with dilation of the left lateral ventricle (Figure 5), and a paradoxical midline shift towards the left side (Figure 6). The timeline of events is mentioned in Table 2.

**Table 2:** Timeline of the events.

Dates	Events
March 19, 2008	Episode of seizure, followed by right hemiparesis.
April 2, 2008 to August 19, 2008	Treatment taken at Ayurveda hospital.
September 7, 2018	Severe anaemia secondary to chronic malaria, treatment taken at government hospital.
September 15, 2018	MRI brain was done, diagnosed with DDMS.
December 7, 2022	Episode of seizure.
December 9, 2022	Reported for physiotherapy treatment.
December 10, 2022	MRI brain and CT scan was done.

## Discussion

DDMS can be categorized as either primary or congenital and secondary or acquired. Congenital DDMS usually does not have obvious etiological factors. Symptoms usually appear at birth or shortly after. In the secondary type, the symptoms are linked to an injury to the central nervous system that happens during pregnancy or afterwards. The etiological factors reported till now are trauma, ischemic and haemorrhagic conditions, infection (cerebral malaria and encephalitis), vascular abnormalities such as coarctation of the midaortic arch, internal carotid hypoplasia or agenesia, and a reduced or absent middle cerebral artery [6,7].

In the present case, symptoms first appeared in early childhood and were of sudden onset without any history of trauma or illness. However, at the age of 15 years, the patient was diagnosed with severe anaemia, which was secondary to chronic malaria. A brain MRI was done, which led to the diagnosis of DDMS. One of the limitations is that no investigations were done during the first episode, and hence the obvious aetiology is unknown.

CT, or magnetic resonance imaging, is the gold standard of investigations for the diagnosis of DDMS. The underlying pathologic processes, nature, and extent differ widely. In the largest series of DDMS studies, 26 patients with a mean age of 11 concluded male sex dominance (73.5%) and left hemisphere involvement (69.2%) [8]. Symptoms that are commonly seen are recurrent seizures, facial asymmetry, and mental retardation [9]. The present case reports a female patient with right hemiparesis without any positive antenatal history and no history of recurrent seizures. Episodes occurred between the ages of 3 and 17, and the patient was not on any medication during that interval of time. The clinical picture includes pure motor hemiparesis on the right side with maximum involvement of the hand and no obvious facial asymmetry or mental retardation, unlike the clinical pictures described in the literature [10].

None of the previous studies have reported the functional status of the patients, which is equally important as that of diagnosis, as two patients with the same diagnosis may have an entirely different clinical presentation and hence may differ in prognosis. The present case exhibits classical features of the acquired type of cerebral hemiatrophy involving the left cerebral hemisphere. This case has deviated from the usual presentation of male predominance and recurrent seizures. The patient is independent in her daily activities despite the severe tenodesis effect seen at hand. The prognosis is good when hemiparesis occurs after the age of two years and there is an absence of prolonged or recurrent seizures [11]. Management is mainly symptomatic [11]. With a mixed picture of prognosis reported in the literature, this patient may have a better functional outcome with physiotherapy treatment.

## Conclusions

This is one of the rare cases of DDMS, standing out prominently due to features of single episodes of seizure at three years of age and then directly at 17 years of age referred for the physiotherapy management of infantile hemiparesis, contradictory to the reported cases of DDMS where repeated seizure is a common finding.
